# Pharmacological Characterization of a 5-HT_1_-Type Serotonin Receptor in the Red Flour Beetle, *Tribolium castaneum*

**DOI:** 10.1371/journal.pone.0065052

**Published:** 2013-05-31

**Authors:** Rut Vleugels, Cynthia Lenaerts, Arnd Baumann, Jozef Vanden Broeck, Heleen Verlinden

**Affiliations:** 1 Department of Animal Physiology and Neurobiology, Zoological Institute, KU Leuven, Leuven, Belgium; 2 Institute of Complex Systems (ICS4), Research Centre Jülich, Jülich, Germany; University of Rouen, France

## Abstract

Serotonin (5-hydroxytryptamine, 5-HT) is known for its key role in modulating diverse physiological processes and behaviors by binding various 5-HT receptors. However, a lack of pharmacological knowledge impedes studies on invertebrate 5-HT receptors. Moreover, pharmacological information is urgently needed in order to establish a reliable classification system for invertebrate 5-HT receptors. In this study we report on the molecular cloning and pharmacological characterization of a 5-HT_1_ receptor from the red flour beetle, *Tribolium castaneum* (Trica5-HT_1_). The Trica5-HT_1_ receptor encoding cDNA shows considerable sequence similarity with members of the 5-HT_1_ receptor class. Real time PCR showed high expression in the brain (without optic lobes) and the optic lobes, consistent with the role of 5-HT as neurotransmitter. Activation of Trica5-HT_1_ in mammalian cells decreased NKH-477-stimulated cyclic AMP levels in a dose-dependent manner, but did not influence intracellular Ca^2+^ signaling. We studied the pharmacological profile of the 5-HT_1_ receptor and demonstrated that α-methylserotonin, 5-methoxytryptamine and 5-carboxamidotryptamine acted as agonists. Prazosin, methiothepin and methysergide were the most potent antagonists and showed competitive inhibition in presence of 5-HT. This study offers important information on a 5-HT_1_ receptor from *T. castaneum* facilitating functional research of 5-HT receptors in insects and other invertebrates. The pharmacological profiles may contribute to establish a reliable classification scheme for invertebrate 5-HT receptors.

## Introduction

Biogenic amines play an important role in very diverse physiological processes and behaviors. In insects, the six major biogenic amines are serotonin (5-hydroxytryptamine, 5-HT), dopamine, tyramine, octopamine, acetylcholine and histamine. 5-HT is known to play a crucial role in the regulation of important processes in most, if not all, animal phyla. Alterations in 5-HT neurotransmission are associated with several human disorders, such as migraine, depression, schizophrenia and anxiety [1]. Normal human processes, such as sleep, mood level, appetite, sexual activity and learning abilities are also modulated by 5-HT. In insects, 5-HT signaling controls nutrition [2], modulation of heart rate [3], secretory processes in the salivary gland [4]–[7], development [8], circadian rhythms and sleep regulation [9], [10], aggression [11], behavioral gregarization in locusts [12], [13], phototactic behavior in honeybees [14] and learning and memory in fruit flies [15], [16].

To mediate such a variety of processes, 5-HT acts through multiple 5-HT receptor types. In vertebrates, 5-HT receptors are divided in seven main classes. Six of these are G protein-coupled receptors (GPCRs) and the sole exception, 5-HT_3_, is a ligand-gated ion channel. GPCRs play a vital role in many essential signaling pathways in all eukaryote organisms. The vertebrate 5-HT GPCRs (5-HT_1,2,4–7_) were classified based on their sequence similarities, gene organization, downstream signaling pathways and pharmacological properties [17]–[20]. 5-HT_1_ and 5-HT_5_ receptors couple preferentially to G_i/o_ proteins and thus inhibit cyclic AMP (cAMP) synthesis. 5-HT_2_ receptors couple preferentially to G_q/11_ proteins which cause an increase in cytosolic Ca^2+^ levels. 5-HT_4_, 5-HT_6_ and 5-HT_7_ receptors are all preferentially linked to G_s_ proteins and promote cAMP production.

The major classes, 5-HT_1_, 5-HT_2_, and 5-HT_6_, probably evolved from a primordial 5-HT receptor over 750 million years ago. The 5-HT_5_ and 5-HT_7_ receptor classes diverged from 5-HT_1_ 650 to 700 million years ago [21], [22]. Since these events even predate the estimated divergence of protostomes and deuterostomes about 600 to 650 million years ago [23], the invertebrate and vertebrate serotonergic systems are believed to possess roughly the same main receptor classes [21], [24]. However, evolution allowed further differentiation in various subtypes within each main class, and these subtypes are believed to have evolved independently in vertebrates and invertebrates [21], [25]. Thus far, only four types of 5-HT receptors are characterized in insects, namely 5-HT_1A_, 5-HT_1B_, 5-HT_2_, and 5-HT_7_ [8], [14], [26]–[29]. Classification of invertebrate 5-HT receptors according to the existing vertebrate classes is mainly based on well conserved amino acid sequences and activated second-messenger systems for 5-HT receptors across all species. On the other hand, pharmacological profiles from insect (and other invertebrate) receptors seem to differ significantly from those of vertebrates [25]. Since only very few data about pharmacological properties of invertebrate 5-HT receptors are available, there is no general classification system for invertebrate 5-HT receptors based on pharmacological properties yet. This explains the need for detailed pharmacological studies on insect and other invertebrate 5-HT receptors.

In the present study, we will discuss the characterization of a 5-HT_1_ receptor from the red flour beetle, *Tribolium castaneum* (Trica5-HT_1_). The genome of *T. castaneum* has been completely sequenced (*Tribolium* Genome Sequencing Consortium) [30]. Use of annotation software led to the discovery of twenty *Tribolium* genes that code for putative biogenic amine GPCRs. All these proteins have orthologues in *Drosophila melanogaster* and *Apis mellifera* [31]. Four receptors could be assigned as putative 5-HT receptors based on sequence similarity to 5-HT receptors of *D. melanogaster* and *A. mellifera* [8], [14], [26]–[28], [31]. After cloning the Trica5-HT_1_ cDNA, we analyzed its tissue distribution by quantitative real-time PCR (qRT-PCR) and elucidated its downstream signaling pathway. In cells expressing Trica5-HT_1_, application of 5-HT inhibited NKH-477 (a water-soluble forskolin analog) stimulated cAMP synthesis. The pharmacological profile of the receptor was established after application of several synthetic 5-HT receptor agonists and antagonists. These results will facilitate future *in vivo* studies aiming to unravel the contribution of individual 5-HT receptors to the animals’ physiology and behavior.

## Materials and Methods

### Animal Rearing Conditions

Beetles were reared in a dark incubator at 30°C on wheat flour and brewer’s yeast in Petri dishes. Adult beetles were sexed based on the presence of a small patch of short bristles on the inside of the first pair of legs in males, according to the *T. castaneum* rearing protocol (http://bru.gmprc.ksu.edu/proj/tribolium/wrangle.asp) [32].

### Cloning of Trica5-ht1 and Construction of pcTrica5-ht1 Expression Vector

The full length sequence encoding the receptor was amplified with PCR using whole body *T. castaneum* cDNA, Taq polymerase (REDTaq®ReadyMix™PCR Reaction Mix, Sigma-Aldrich), and 10 µM of sense primer 5′-ATGGGGACAGTAAATAATCCCTCCTG-3′ and antisense primer 5′-TTATCTAATTTTGCCCGAGCGG-3′ (Sigma-Aldrich). Primers were designed based on sequences available in Beetlebase (Tcas_3.0; http://www.beetlebase.org/) [33] released by the Human Genome Sequencing Center. PCR started with initial denaturation for 2 min at 95°C, followed by 35 cycles of [30 s at 94°C, 30 s at 62°C, 2 min at 68°C], followed by final elongation for 2 min at 68°C. Amplification products were run on a 1.2% agarose gel and purified with the GenElute™ Gel extraction Kit (Sigma–Aldrich). The DNA fragments were cloned into a pcDNA3.1/V5-His-TOPO®TA expression vector via TA TOPO cloning (Invitrogen) and transformed into One Shot TOP10 chemically competent *Escherichia coli* cells (Invitrogen). Bacteria were grown according to the protocol recommended by the kit. Plasmids were isolated via the GenElute™ HP Plasmid Miniprep kit (Sigma-Aldrich) and DNA sequences were determined by means of the ABI PRISM 3130 Genetic Analyzer (Applied Biosystems) following the protocol outlined in the ABI PRISM BigDye Terminator Ready Reaction Cycle Sequencing Kit (Applied Biosystems). Bacterial cells known to contain the correct receptor insert were grown at large scale in 100 ml Luria–Bertani broth medium. The expression vectors were subsequently isolated from these cells using the EndoFree Plasmid Maxi Kit (Qiagen) according to the protocol recommended by the kit.

### qRT-PCR Study of Transcript Levels

For determination of expression levels of the receptor, tissues from sexually mature *T. castaneum* were dissected in phosphate buffered saline (PBS) (NaCl 137 mM, KCl 2.7 mM, Na_2_HPO_4_ 10 mM, KH_2_PO_4_ 1.76 mM; pH 7.2) and snap-frozen in liquid nitrogen. For all samples, tissues of at least fifteen animals were pooled. Tissues were homogenized and RNA was extracted using the RNAqueous Micro Kit (Ambion) according to the protocol recommended by the kit. The protocol included an additional DNase treatment to digest remaining DNA. Total RNA was reverse transcribed into cDNA using SuperScriptIII reverse transcriptase (Invitrogen) as recommended by the manufacturer, and diluted ten-fold prior to use. Transcript levels were quantified using the Fast Sybr Green assay kit (Applied Biosystems) in a StepOne Plus detection system (ABI Prism, Applied Biosystems). Primers (sense primer 5′-GCCCTCTGGCTGGGCTAT-3′ and antisense primer 5′-CGGGTTGAAGATCGTGTAAATGA-3′) (Sigma-Aldrich) were used in final concentrations of 500 nM. Other conditions were as recommended by the manufacturer. Reactions were run in duplicate and incubated for 2 min at 50°C, followed by 10 min at 95°C, followed by 40 cycles of [15 s at 95°C and 1 min at 60°C]. The specificity of the PCR products was assessed generating a dissociation curve (95°C for 15 s, 60°C for 1 min, and increase in temperature in 0.7°C increments from 60°C to 95°C). Agarose gel electrophoresis of the PCR products confirmed the presence of a single band of the expected size and sequencing confirmed their identity. The relative quantity of target cDNA was quantified using the ΔΔC_T_-method including normalization to a calibrator on all PCR plates and an endogenous control. From a list of seven housekeeping genes (Table S1; [34]), the combination of genes for this endogenous control was determined using GeNorm [35]. Expression was most stable for RPs3 (ribosomal protein 3) and RPs18 with respect to sex and tissue and these transcripts were thus selected for further use as endogenous controls (results not shown).

### Cell Culture and Transfection

General binding studies were performed in Chinese hamster ovary (CHO) WTA11 cells stably coexpressing apoaequorin and the promiscuous G_α16_. This allowed us to measure the pharmacology independent of the downstream signaling. CHO-PAM28 cells stably expressing apoaequorin, but not the promiscuous G_α16_, and human embryonic kidney cells (HEK) 293 cells were used to measure downstream signaling via Ca^2+^ and cAMP, respectively. CHO cell lines were provided by Prof. Marc Parmentier (University of Brussels, Belgium) and Dr. Michel Detheux (Euroscreen S.A., Belgium). HEK293 cells were a gift from Prof. Arnd Baumann (Research Centre Jülich, Germany.

All cells were cultured in Dulbecco’s Modified Eagles Medium nutrient mixture F12-Ham (DMEM/F12) (Invitrogen) supplemented with 1% penicillin/streptomycin (10000 units/ml penicillin and 10 mg/ml streptomycin in 0.9% NaCl) (Invitrogen) to prevent bacterial contamination of gram-positive and gram-negative bacteria, respectively. For CHO-WTA11 cells, 250 µg/ml zeocin (Invitrogen) was added and for CHO-PAM28 cells, 5 µg/ml puromycin dihydrochloride (Invitrogen) was added. Puromycin and zeocin were initially used to select for cells stably expressing apoaequorin (CHO-PAM28) [36], or both apoaequorin and G_α16_ (CHO-WTA11 cells) [37] and are thus still used as additional antibiotics in the appropriate screens. For CHO cells, the medium was supplemented with 10% fetal calf serum (inactivated at 65°C) (Sigma-Aldrich). For HEK293 cells, the medium was supplemented with 2% Ultroser G serum substitute (Pall Life Sciences).

Cells were cultured *in vitro* as monolayers at 37°C, 5% CO_2_ and high relative humidity, and subcultivated twice a week. For transfection of CHO cells, 2.5 ml Opti-MEM®I (Invitrogen), 5 µg plasmid DNA and 12.5 µl Plus^TM^reagent (Invitrogen) were mixed, stored at room temperature for 5 min and next repleted with 30 µl Lipofectamine™LTX (Invitrogen). After 30 min incubation at room temperature, this transfection mixture was added dropwise to the cells together with 5 ml medium. HEK293 cells were cotransfected with receptor construct (4 µg) and CRE-luciferase construct (2 µg), consisting of the open reading frame of the reporter gene, luciferase, downstream of a multimerized cAMP-response-element (CRE_6x_) [38], [39]. All transfections were performed in T75 flasks with a confluency of about 60 percent. Cells were grown overnight, after which 15 ml of medium was added for an additional overnight incubation period.

### Aequorin-luminescence Assay

The transfected CHO cells were detached with phosphate buffered saline (PBS) containing 0.2% EDTA and collected in DMEM/F-12 (without phenol red, with L-glutamine and 10 mM HEPES) (Gibco). The amount of viable cells was determined using the NucleoCounter NC-100+TM (Chemometic). Cells were pelleted for 4 min at 800 rpm at room temperature and resuspended in BSA-medium (DMEM/F12 without phenol red, with L-glutamine and 10 mM HEPES, supplemented with 0.1% bovine serum albumin) to a concentration of 5×10^6^ cells/ml. Coelenterazine H (Invitrogen) was added to a final concentration of 5 µM, and cells were gently shaken for 4 h at room temperature in the dark. After a 10-fold dilution in BSA-medium, cells were incubated another 30 min. The pharmacological ligands were dissolved in BSA-medium. For agonists, 50 µl containing the final ligand concentration was added to appropriate wells of a 96-well plate. For antagonists, 25 µl of the antagonist solution was supplemented with 25 µl of a 5-HT solution. Receptor activity was measured as the light emission after adding 50 µl of the cell suspension. Light emission was measured for 30 s using a Mithras LB940 (Berthold Technologies). Subsequently, cells were lysed with Triton X-100 (0.1% in BSA-medium) and light emission was recorded for another 8 s. BSA-medium was used as a negative control. Light emission from each well was calculated relative to the total response (ligand+Triton X-100) using the output file of Mikrowin2000 software (Mikrotek). Further analysis was done in Graphpad Prism 5.

### Luciferase Reporter-gene Assay

Cotransfected HEK293 cells were detached and the amount of viable cells was determined as described for CHO cells. Cells were pelleted for 4 min at 800 rpm at room temperature and resuspended in DMEM/F12 medium (without phenol red, with L-glutamine and 10 mM HEPES) containing 200 µM 3-isobutyl-1-methylxanthine (IBMX, Sigma-Aldrich) to a concentration of 10^6^ cells/ml. The pharmacological ligands were dissolved in DMEM/F12 medium without phenol red containing 200 µM IBMX. The water-soluble forskolin analog NKH-477 was added to a concentration of 20 µM to measure *Trica5-ht1*-mediated effects on cellular cAMP signaling. In each well of a 96-well plate, 50 µl of ligand suspension and 50 µl of cell suspension were dispensed. After incubation for 3–4 h in a CO_2_ incubator at 37°C, 100 µl of SteadyLite Plus (Perkin-Elmer) was added to each well and the plate was gently shaken for 15 min in the dark. Light emission was measured for 5 s per well using a Mithras LB940 (Berthold Technologies). Medium containing IBMX was used as a negative control. Data were analyzed as described for CHO cells.

### Drugs

The pharmacological ligands 3-hydroxytyramine (dopamine) hydrochloride, 5-carboxamidotryptamine maleate (5-CT), 5-HT hydrochloride (5-HT), 5-methoxytryptamine (5-MT), (±)-8-hydroxy-2-(dipropylamino)tetralin hydrobromide (8-OH-DPAT), α-methylserotonin maleate (αm-5-HT), (+)-butaclamol hydrochloride, ketanserin (+)-tartrate, methiothepin mesylate, methysergide maleate, mianserin hydrochloride, prazosin hydrochloride, DL-octopamine hydrochloride, SB-269970 hydrochloride, tyramine hydrochloride, WAY-100635 maleate (WAY = N-{2-[4-(2-methoxyphenyl)-1-piperazinyl]-ethyl}-N-(2-pyridinyl) cyclohexanecarboxamide), and yohimbine hydrochloride were purchased from Sigma-Aldrich.

## Results

### Cloning and Sequence Analysis of Trica5-ht1

**Figure 1:**
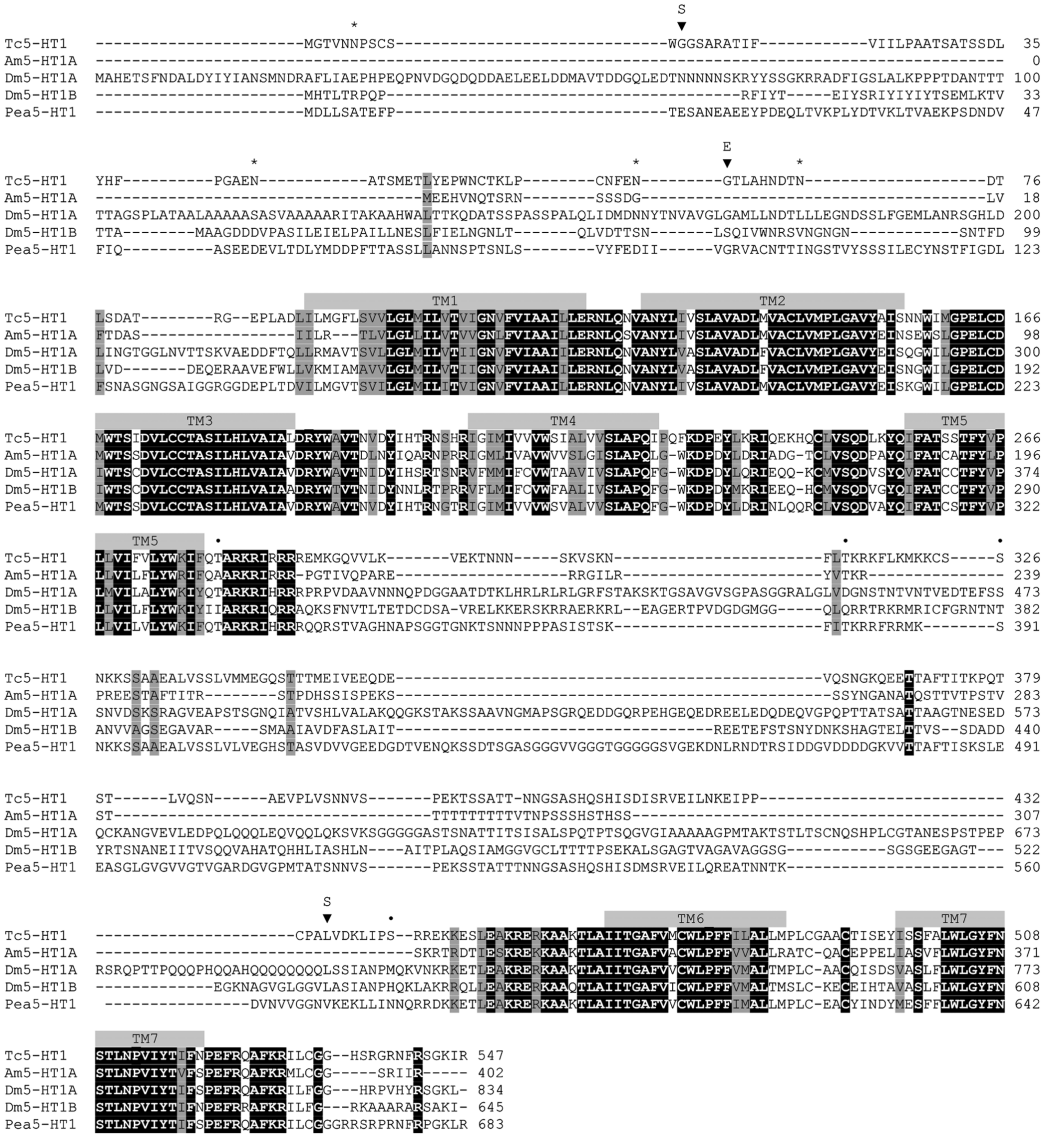
Amino acid sequence alignment of *T. castaneum* 5-HT_1_ sequence (Trica5-HT_1_, GenBank accession no. KC196076). Alignment against sequences of orthologous receptors from *Apis mellifera* (Am5-HT_1A_, no. CBI75449), *Drosophila melanogaster* (Dm5-HT_1A_, no. CAA77570 and Dm5-HT_1B_, no. CAA77571), and *Periplaneta americana* (Pea5-HT_1_, no. CAX65666). Identical residues between the receptors are shown as white characters against black background. Conservatively substituted residues are shaded. Putative transmembrane domains are indicated by grey bars (TM1-7). Dots indicate putative phosphorylation sites for PKC, and stars indicate putative N-linked glycosylation sites. Inverted triangles indicate differences between the current sequence derived from cloned cDNA and the annotated sequence from Beetlebase.

A cDNA fragment encoding a 5-HT_1_ receptor from *T. castaneum* was amplified by PCR. The open reading frame of Trica*5-ht1* contains 1,644 nucleotides (Figure S1) encoding the Trica5-HT_1_ protein of 547 amino acids (Figure 1) with a calculated molecular weight of 60.8 kDa. Transmembrane topology prediction revealed the presence of seven putative transmembrane domains (TM1-7), characteristic of all GPCRs. Consensus motifs for N-linked glycosylation (N-x-[S/T]) are found in the extracellular N-terminus, and consensus sites for phosphorylation by protein kinase C (PKC) ([S/T-x-[R/K]) are located within the third intracellular loop (Figure 1). The C^7.69^ residue (numbering according to the Ballesteros-Weinstein system [40]) in the intracellular C-terminus is a putative palmitoylation site. The large third intracellular loop and the short intracellular C-terminal region are consistent with other known 5-HT_1_ and biogenic amine receptors that couple via G_i_. Other typical biogenic amine and 5-HT receptor characteristics are present as well. The DRY tripeptide (D^3.49^R^3.50^Y^3.51^) located in the second intracellular loop is the key to the conformational changes necessary for receptor activation [41]. The combination of the D^3.32^ in TM3 with the conserved W^7.40^ in TM7 is considered a unique fingerprint for biogenic amine and trace amine GPCRs. The charged D^3.32^ residue is thought to interact with the protonated amine moiety of amine ligands [42], [43]. As in other 5-HT receptors, Trica5-HT_1_ typically has a conserved group of hydrophobic amino acids (W^3.28^, F^5.47^, W^6.48^, F^6.51^, F^6.52^, W^7.40^, Y^7.43^) that form the hydrophobic ligand-binding pocket within the tertiary structure. This binding pocket may be stabilized by a disulfide bridge formed between C^2.55^ in TM2 and C^3.25^ in extracellular loop 1 [42], [44], [45]. In addition, the consensus sequence of non-peptide receptors in TM6, F^6.44^-x-x-x-W^6.48^-x-P^6.50^, is followed by a pair of Phe residues (F^6.51^ and F^6.52^) unique to aminergic receptors. Also the N^7.49^P^7.50^-x-x-Y^7.53^ motif in TM7 is conserved, which may participate in agonist mediated receptor sequestration and resensitization [46].

BLASTx (http://blast.ncbi.nlm.nih.gov/blast/) searches indicate similarities of the receptor to other insect 5-HT_1_ receptors (Pea5-HT_1_, 60% identity; Am5-HT_1A_, 55% identity; Dm5-HT_1A,_ 46% identity; Dm5-HT_1B_, 42% identity). When compared to the computationally predicted sequence in Beetlebase [31], a stretch of 75 bp is missing in our cloned receptor cDNA sequence. Since this stretch is flanked by splice sites and not present in cDNA sequences of other species, it is presumed to be an intron. After multiple sequencing runs on different cDNA samples, also five single base mismatches (three silent and two missense mutations) were found between the amplified and the annotated sequence. Furthermore, two consecutive nucleotides were different, resulting in an amino acid change (Figure S1).

### Transcript Level Study

**Figure 2:**
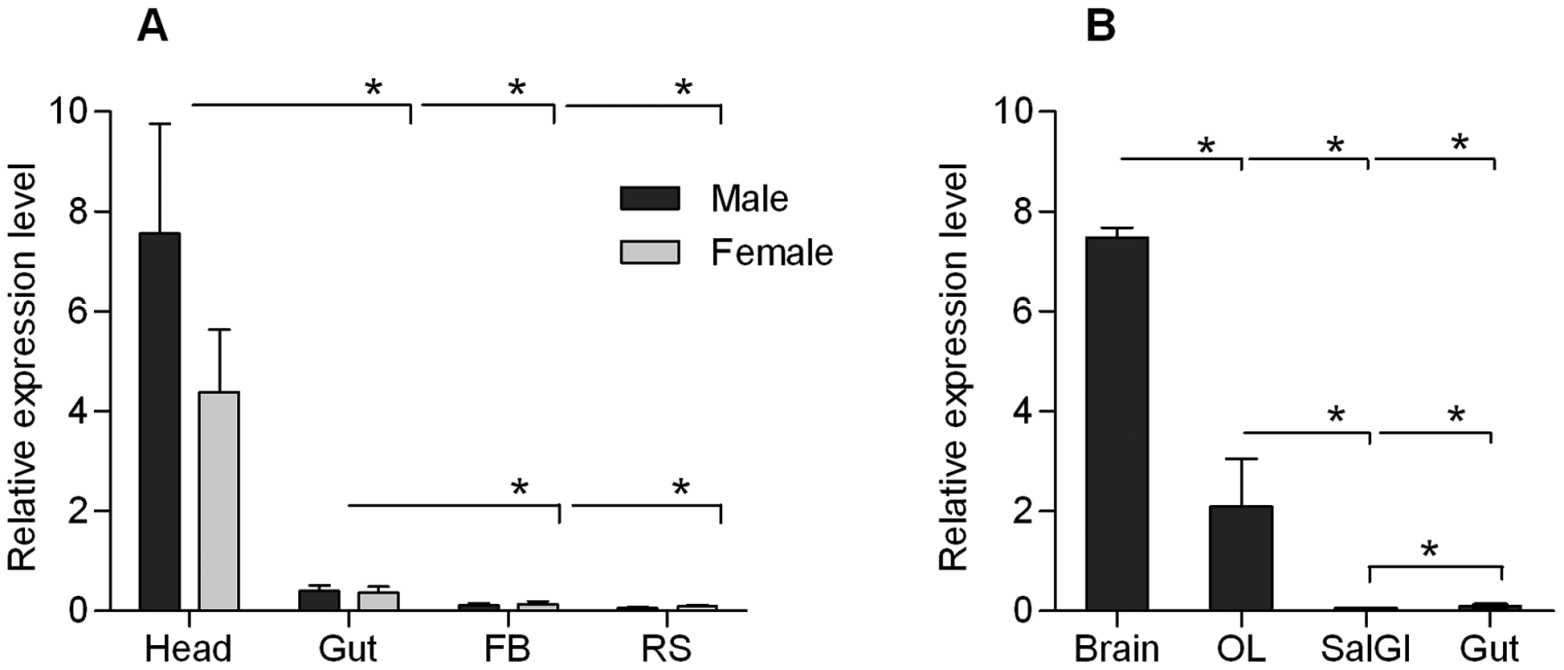
Expression profile of transcripts encoding Trica5-HT_1_ in sexually mature beetles. The data represent mean values of (A) three independent samples of 30×heads, 50×guts, 20×fat body and 50×reproductive system; and (B) three independent samples of 15 beetles each; run in duplicate ± SEM, normalized relative to RPs3 (ribosomal protein 3) and RPs18 transcript levels. Statistically significant differences are indicated by asterisks above the bars (p≤0.05) (Kruskal-Wallis, IBM SPSS Statistics 20). Abbreviations: FB, fat body; RS, reproductive system; Brain, brain without the optic lobes; OL, optic lobes; SalGl, salivary glands.

An initial tissue distribution screen of Trica5-HT_1_ mRNA in sexually mature beetles was performed using qRT-PCR (Figure 2A). Transcripts of the Trica*5-ht1* gene were detected in considerable amounts in the head and the gut, consistent with the prevalence of 5-HT in central nervous system (CNS) and the digestive tract of most animal species. However, expression in the head appeared significantly more abundant. Differences between males and females were not significant. In the fat body and the reproductive tract of sexually mature beetles, only very low levels of receptor transcript were observed. When transcript levels in the brain (without the optic lobes) and the optic lobes were measured separately, expression in both tissues seemed significantly higher than in the gut (Figure 2B). Moreover, expression in the brain (without the optic lobes) was 3.5 times higher compared to the optic lobes. Although there are indications for 5-HT receptor expression in the salivary gland of some insect species, we detected almost no Trica*5-HT_1_* transcripts in the salivary gland of adult beetles.

### Pharmacological Characterization and Downstream Signaling Properties of Trica5-HT1

**Figure 3:**
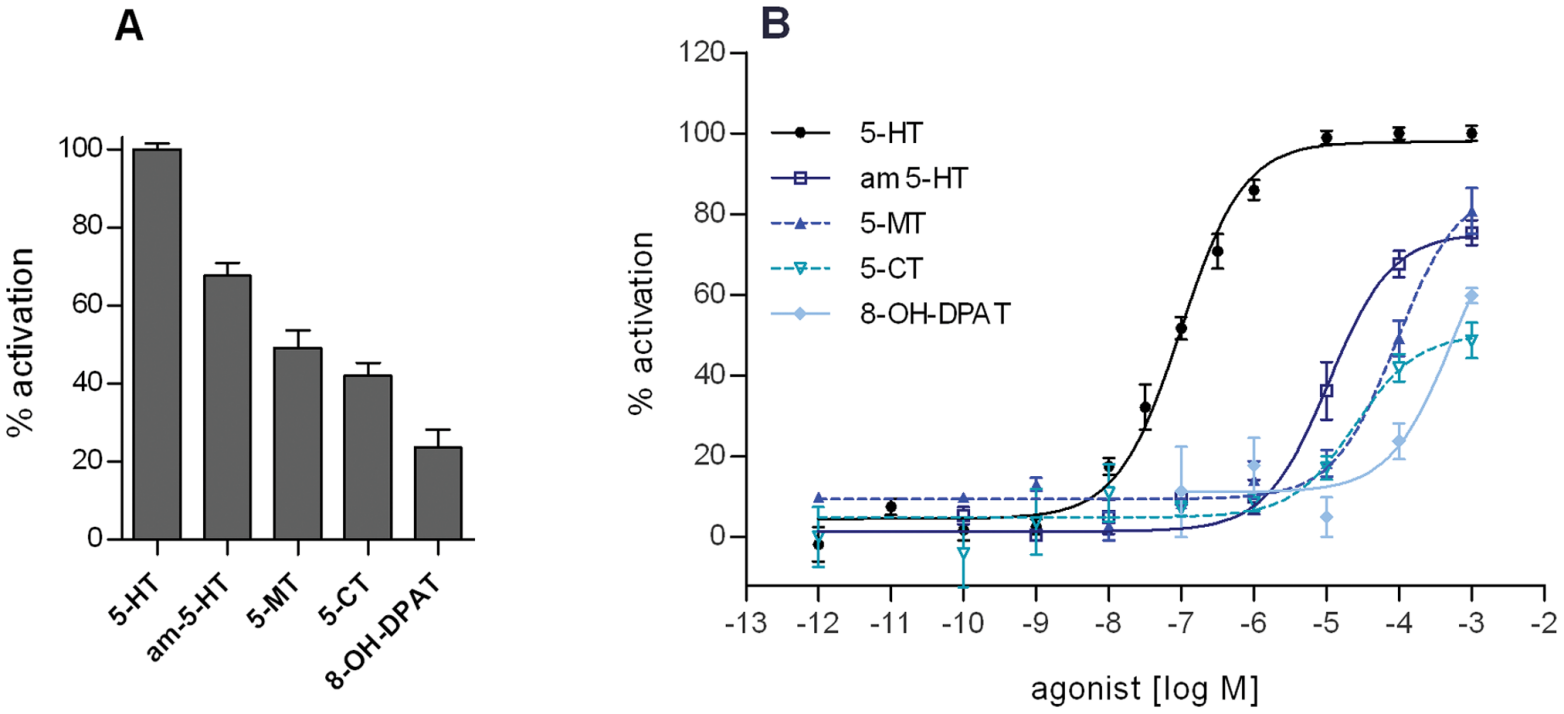
Effect of various agonists on Trica5-HT_1_ in CHO-WTA11 cells. (A) Receptor activation after stimulation with 100 µM of agonist, shown as the percentage of activation achieved with 100 µM of 5-HT (set at 100%). (B) Dose-dependent activation of Trica5-HT1 with synthetic 5-HT receptor agonists, shown as the percentage of activation achieved with 1 mM 5-HT (maximum response = 100%). Cells treated with BSA-medium only were used to define the basal level of 0%. Data represent the mean ± SEM of (A) three independent measurements (each performed in duplicate) and (B) seven independent measurements (each performed in duplicate) for 5-HT, or four independent measurements (three performed in triplicate, one performed in duplicate) for the synthetic agonists αm-5-HT, 5-MT, 5-CT and 8-OH-DPAT.

In order to pharmacologically characterize Trica5-HT_1_, we used CHO-WTA11 cells stably expressing apoaequorin and the promiscuous G_α16_ subunit. No 5-HT evoked signal was observed in non-transfected cells or in cells transfected with an empty vector (results not shown). Significant responses were obtained when cells expressing Trica5-HT_1_ were incubated with 100 µM 5-HT or the 5-HT receptor agonists αm-5-HT, 5-CT, 5-MT and 8-OH-DPAT (Figure 3A). The dose-response relationship for 5-HT and these synthetic agonists was examined at concentrations ranging from 1 pM to 1 mM (Figure 3B). The resulting sigmoidal dose-response curve of 5-HT shows receptor activation in a dose-dependent and saturable manner. Half-maximal activation (EC_50_) was achieved at 5-HT concentrations of 95.15 nM (logEC_50_ = −7.01±0.043, mean ± SEM). The maximal response was attained at 5-HT concentrations of ≥10 µM. Since the efficacy achieved by any agonist depends on the number of receptors expressed, we measured a dose-response curve for 5-HT in every experiment and normalized all agonist effects to the maximum 5-HT response, set at 100% (Figure 3B). Surprisingly, the most potent agonist was the mammalian 5-HT_2_ receptor agonist, αm-5-HT, with an EC_50_ value of 10.74 µM (logEC_50_ = −4.97±0.18, mean ± SEM). However, this is more than 100-fold less potent than 5-HT itself. Also the 5-HT analog, 5-CT, a selective agonist for mammalian 5-HT_1_ and 5-HT_7_ receptors, and the non-selective 5-HT receptor agonist, 5-MT, acted as partial agonists in a dose-dependent manner. 5-CT was more potent but had a lower efficacy than 5-MT. The EC_50_ values for all agonists are shown in Table 1. 8-OH-DPAT, a partial and selective agonist for mammalian 5-HT_1_ and 5-HT_7_ receptors evoked responses only at concentrations ≥100 µM. The biogenic amines, dopamine, octopamine and tyramine did not generate any detectable responses at concentrations ≤100 µM (results not shown).

**Table 1:** EC_50_ values of agonists for Trica5-HT_1_ receptor activation in CHO-WTA11 cells.

agonist	EC_50_ (µM)	logEC_50_ (mean ± SEM)
5-HT	0.095	−7.01±0.043
αm-5-HT	10.74	−4.97±0.18
5-CT	24.72	−4.61±0.17
5-MT	91.84	−4.04±0.12
8-OH-DPAT	551.0	−3.26±0.59

**Figure 4:**
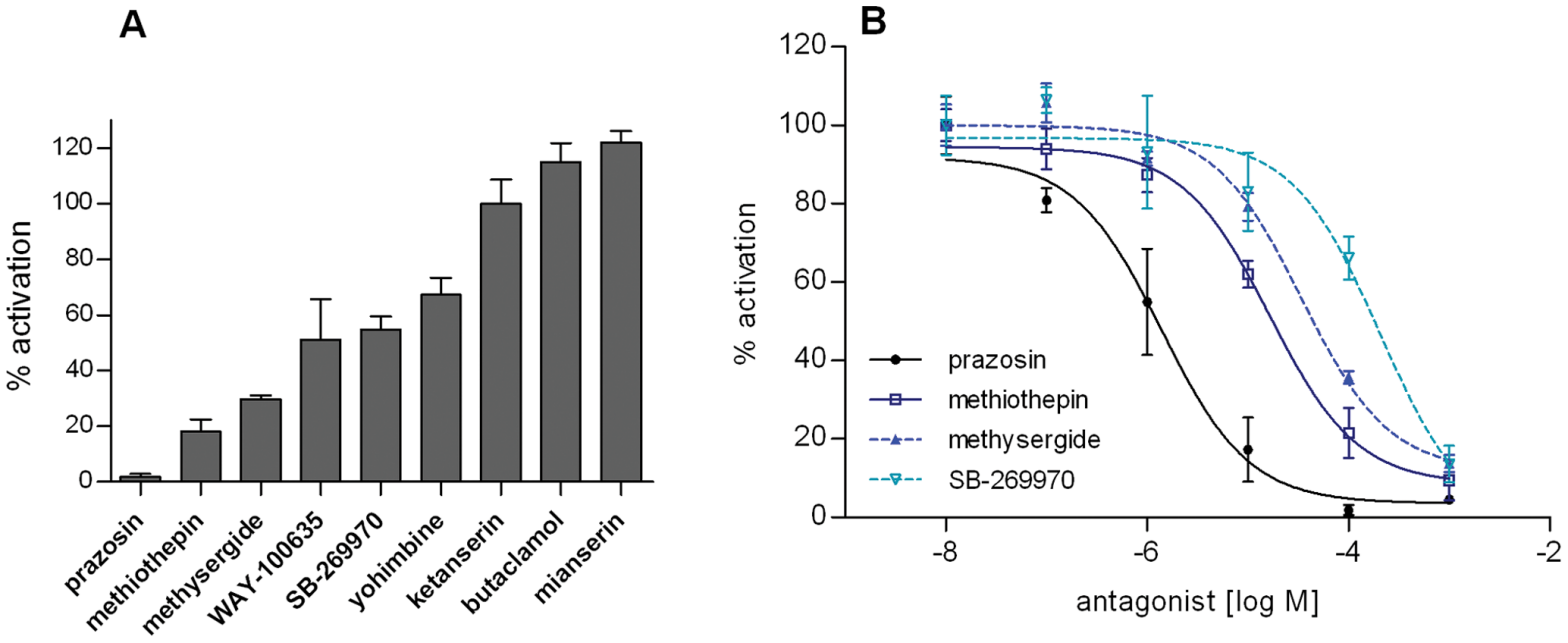
Effect of various antagonists on 5-HT mediated activation of Trica5-HT_1_ in CHO-WTA11 cells. (A) Effect of antagonists (100 µM) on 5-HT (100 nM) mediated receptor activation. Receptor activation is shown as the percentage of activation achieved with 100 nM of 5-HT (∼ EC_50_ value) (set at 100%). (B) Dose-dependent effect of 5-HT receptor antagonists on 5-HT (100 nM) mediated receptor activation. Receptor activation is shown as the percentage of activation achieved with 10 nM of antagonist (set at 100%). Cells treated with BSA-medium only were used to define the basal level of 0%. Data represent the mean ± SEM of (A) three independent measurements (each performed in duplicate) and (B) two (prazosin and methysergide) or three (methiothepin and SB-269970) independent measurements (each performed in triplicate).

**Figure 5:**
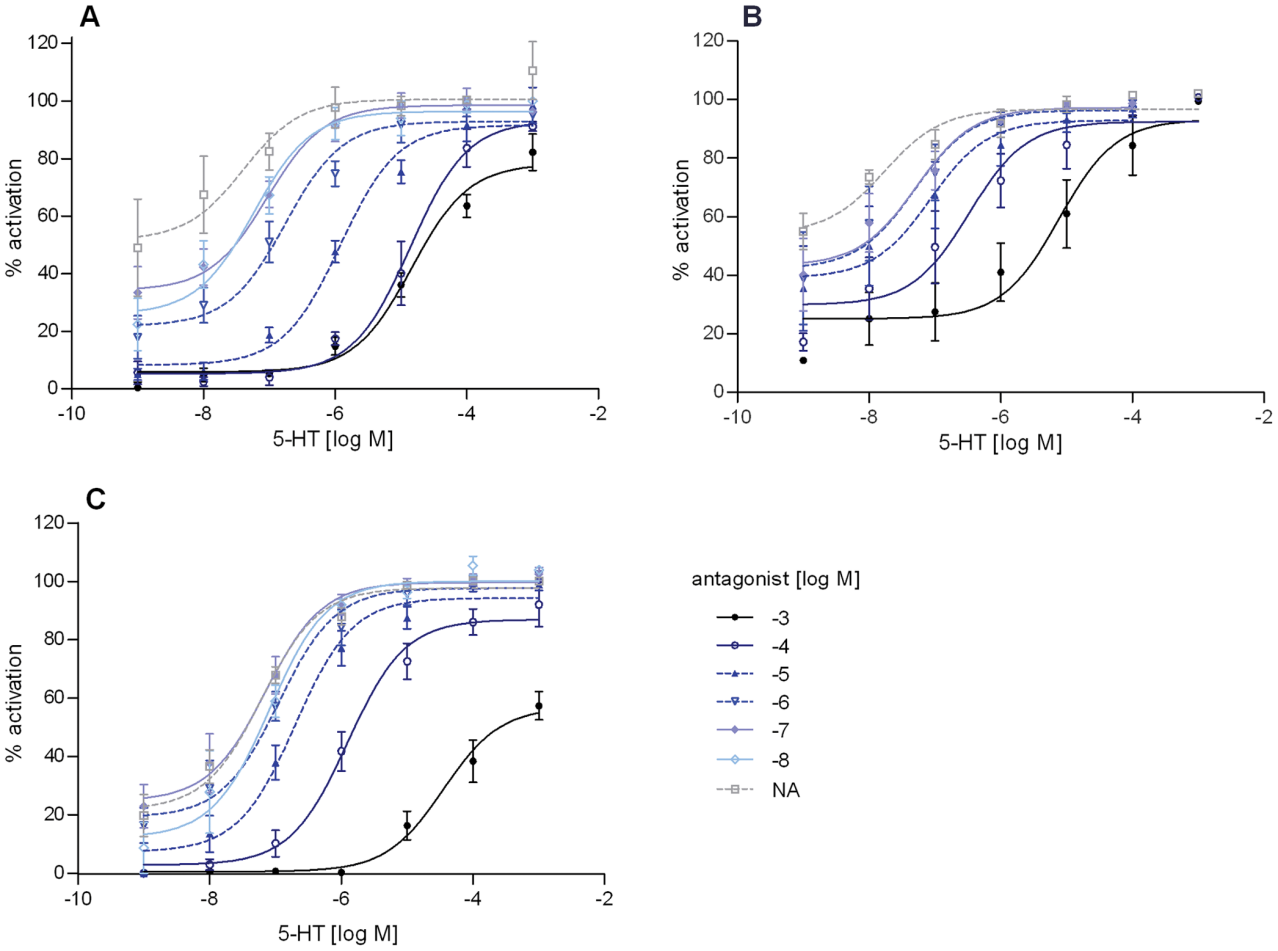
Effect of 5-HT on Trica5-HT_1_ in presence of different concentrations of antagonist. Dose-dependent activation of Trica5-HT_1_ was measured in CHO-WTA11 cells with 1 nM –1 mM 5-HT in presence of 10 nM –1 mM antagonist: (A) prazosin, (B) methysergide and (C) methiothepin. Receptor activity is shown as the percentage of activation achieved with 1 mM of 5-HT (set at 100%). Cells treated with BSA-medium only were used to define the basal level of 0%. Data represent the mean ± SEM of three independent measurements (each performed in duplicate) for prazosin and methiothepin and two independent measurements (each performed in duplicate) for methysergide. NA, no antagonist.

Potential antagonists were tested by simultaneously applying 5-HT (100 nM) and a high dose of antagonist (100 µM) to Trica5-HT_1_ expressing cells (Figure 4A). In addition, the dose-dependence of the antagonistic effects were measured with antagonist concentrations ranging from 10 nM to 1 mM (Figure 4B). From these experiments, we can conclude that ketanserin and mianserin, two selective antagonists of mammalian 5-HT_2_, and butaclamol, a dopamine receptor antagonist, displayed no detectable inhibition of 5-HT induced responses in cells expressing Trica5-HT_1_. Mianserin and butaclamol even seemed to have some agonistic effects at high concentration (100 µM). On the other hand prazosin, a selective α1-adrenergic receptor antagonist in mammals, was shown to be the most potent antagonist. It decreased the effect of 5-HT on the receptor in a dose-dependent manner with a half maximal inhibitory concentration (IC_50_) of 1.39 µM (logIC_50_ = −5.86±0.18, mean ± SEM). The IC_50_ values for all antagonists are shown in Table 2. Methiothepin and methysergide, two non-selective antagonists of mammalian 5-HT receptors also showed dose-dependent inhibition. Moderate inhibition was achieved with SB-269970, a selective antagonist of mammalian 5-HT_7_. The level of inhibition induced by WAY-100635 didn’t drop below 50% and was variable which might be due to complex effects of the compound on the receptor and/or other targets in the assay. Also yohimbine, known to behave as both agonist and antagonist on some mammalian 5-HT-receptors, showed only about 30% of inhibition, even at concentrations up to 100 µM. The nature of inhibition obtained with the three most potent antagonists: prazosin, methiothepin and methysergide was further examined by studying the dose-response relationship of 5-HT in presence of different concentrations of antagonist (10 nM to 1 mM) (Figure 5). The higher the concentration of antagonist, the higher was the resulting EC_50_ value for 5-HT activity. Since their efficacy didn’t change, these compounds behaved as truly competitive antagonists. We used the Gaddum/Schild plot to compare the inhibitory potencies of the antagonists by their pA2 values (*i.e.* the logarithm of the concentration of antagonist that doubles the amount of 5-HT required for obtaining the same effect) [47]. The pA2 values (± SEM) for prazosin, methiothepin and methysergide were, respectively, 7.18 (±0.13), 6.17 (±0.11) and 5.96 (±0.21), confirming that prazosin has the highest affinity, followed by methiothepin and methysergide.

**Table 2:** IC_50_ values of antagonists for Trica5-HT_1_ receptor inhibition in CHO-WTA11 cells.

antagonist	IC_50_ (µM)	logIC_50_ (mean ± SEM)
prazosin	1.39	−5.89±0.18
methiothepin	16.38	−4.79±0.13
methysergide	33.97	−4.47±0.094
WAY-100635	no clear dose-dependence of inhibition	
butaclamol	204.1	−3.69±0.20
SB-269970	205.4	−3.69±0.25
ketanserin	no inhibition	
yohimbin	no inhibition	
mianserin	no inhibition	

**Figure 6:**
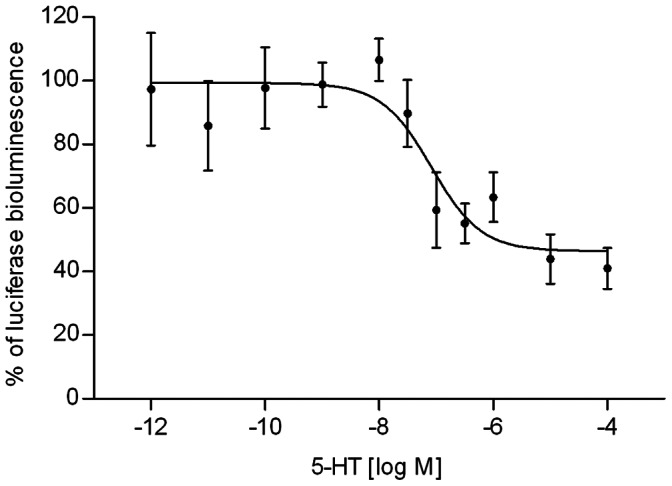
Dose-dependent effect of 5-HT on intracellular cAMP levels in HEK293 cells. The effect of 5-HT (1 pM - 100 µM) on the luciferase bioluminescence in HEK293 cells expressing Trica5-HT_1_ due to changes in intracellular cAMP levels. Receptor activity is shown as the percentage of activation achieved with 10 µM of NKH-477 (set at 100%). Luciferase bioluminescence due to basal intracellular cAMP levels is set at 0%. The data represent the mean ± SEM of four independent measurements (three performed in triplicate, one performed in duplicate).

CHO-PAM28 and HEK293 cells were used to determine the downstream signaling pathway of Trica5-HT_1_. No effect of 5-HT was observed in CHO-PAM28 cells transfected with empty vector or in cells transfected with the receptor. Therefore it can be concluded that Trica5-HT_1_ does not couple via G_q_ to the Ca^2+^ signaling pathway. In HEK293 cells, effects on the cAMP level were examined for 5-HT concentrations ranging from 1 pM to 100 µM. Relative high variation in the data can be explained by assay based variation since cells were not counted when dispensed. Basal levels of cAMP did not significantly change in cells transfected with an empty vector. In Trica5-HT_1_ expressing cells, a dose-dependent decrease in intracellular, NKH-477 stimulated cAMP levels was registered (Figure 6). Half maximal reduction of cAMP was observed at 82.7 nM 5-HT (logIC_50_ = −7.08±0.30), mean ± SEM). Trica5-HT_1_ thus inhibits the cAMP production, probably via the G_i_ protein. Maximal attenuation of cAMP synthesis (±40%) was attained with 5-HT concentrations ≥10 µM.

## Discussion

In the present study, we have characterized Trica5-HT_1_, a 5-HT_1_ receptor of the red flour beetle, *T. castaneum*. The obtained sequence has considerable similarity with orthologous receptors from other invertebrates [14], [27], [29], [48]–[53] and mammals [20], [54]. The sequence contains typical characteristics of 5-HT_1_ receptors, such as a large third intracellular loop, a short C-terminal region, a DRY motif in the second intracellular loop as well as other conserved consensus sequences. Notably, differences between the cloned and annotated nucleotide sequences were uncovered. These included single base mismatches and an intron in the annotated open reading frame (Figure S1). So far, no introns have been reported in the coding regions of vertebrate genes encoding 5-HT_1_ receptors [55]. However, introns were found in the *D. melanogaster* genes encoding Dm5-HT_1A_ and Dm5-HT_1B_ [27].

When studying the Trica5-HT_1_ transcript levels with qRT-PCR, highest expression was observed in the brain (without optic lobes), followed by the optic lobes. More detailed localization studies in other insects also showed receptor expression in the optic lobes as well as other parts of the brain [9], [10], [14], [27], [29]. In *D. melanogaster*, Dm5-HT_1A_ and Dm5-HT_1B_ receptors are expressed in the mushroom bodies [9], [10], the region of the brain involved in learning and memory. Also in the mushroom bodies of *A. mellifera*, high levels of Am5-HT_1A_ were found [14]. Additionaly, Am5-HT_1A_ was found in regions known to be important in visual information processing, such as the optic lobes. Based on behavioral tests 5-HT is assumed to reduce the positive phototactic behavior of honeybees [14]. In *P. americana*, antibodies directed against Pea5-HT_1_, stained some large somata in the *pars intercerebralis* [29]. Thus, expression of Trica5-HT1 in beetle brains may be due to expression in the mushroom bodies and/or cells of the *pars intercerebralis*. Receptor expression in the gut may indicate a role of 5-HT in the regulation of digestion or gut contraction. In humans, for example, as much as 95% of the total 5-HT content may reside in the intestine [56], [57]. It is probable that a substantial release of 5-HT in other animal species, such as *Tribolium*, takes place in the digestive tract as well. In several insects, 5-HT immunoreactive nerve fibers have been localized in different parts of the intestinal tract [58]–[60]. 5-HT was also shown to modulate muscle contractions of the gut in several insects [61]–[66], although it may also act as a paracrine factor and, for example, provoke the release of other factors from neuroendocrine cells. Even then, high 5-HT levels in a given release organ do not necessarily co-incide with the local level of receptor expression, which might explain the relatively low 5-HT_1_ transcript levels in the gut compared to the brain. Although only low transcript levels were observed in the salivary glands of adult beetles, 5-HT also has been shown to be important in salivation in several insect species [4]–[7], [67]. In the cockroach, 5-HT_1_ receptor expression was shown in the salivary gland by RT-PCR and Western blotting [29]. Low expression levels of Trica5-HT_1_ receptors in the salivary gland suggest that possible 5-HT effects on salivation are regulated by other 5-HT receptor subtypes. For example, in the blowfly, *Calliphora vicina*, salivary glands express 5-HT_2_ and 5-HT_7_ receptors [67].

To examine the downstream signaling pathway of Trica5-HT_1_, the receptor was expressed in CHO-PAM28 and HEK293 cells. Since no Ca^2+^ response was measured in CHO-PAM28 cells expressing Trica5-HT_1_, the receptor does not engage G_q_ and the PLC signaling pathway. In HEK293 cells, the receptor was cotransfected with a CRE-luciferase construct to detect changes in intracellular cAMP levels. 5-HT was found to decrease NKH-477-stimulated cAMP synthesis in a dose-dependent manner. In accordance with other 5-HT_1_ receptors, Trica5-HT_1_ couples to G_i/o_ proteins that impair adenlylate cyclase activity. However, when interpolating these experimental data to physiological processes in *Tribolium*, one must be aware of possible discrepancies between effects observed in cultured cell lines and intracellular processes occurring within the *in vivo* context of the organism.

CHO-WTA11 cells were used to investigate the pharmacological characteristics of Trica5-HT_1_. Application of 5-HT to these cells resulted in dose-dependent receptor activities. The EC_50_ value of 95.15 nM is similar to values reported for 5-HT_1_ receptors from other arthropods [14], [27], [29], [52], [53]. Besides 5-HT, three additional ligands caused dose-dependent activation of Trica5-HT_1_, i.e. αm-5-HT, 5-CT and 5-MT. The synthetic agonists were more than a 100-fold less potent than 5-HT and seemed to have a lower efficacy. However it is possible that the agonists did not reach their maximum response although high concentrations (1 mM) were already tested. Similar properties have been reported for other insect 5-HT_1_ receptors [14], [29]. Only a very poor response was observed with 8-OH-DPAT, an agonist for mammalian 5-HT_1_ and 5-HT_7_ receptors. Also on *A. mellifera* and *P. americana* 5-HT_1_ receptors, 8-OH-DPAT acted as a poor agonist [14], [29]. For the antagonists, no inhibition was measured upon application of butaclamol, ketanserin and mianserin. Although WAY-100635 is known as a potent and selective antagonist of mammalian 5-HT_1A_ receptors, no clear dose-dependent effect was observed. In *A. mellifera,* however, WAY-100635 acted as a partial antagonist of Am5-HT_1_ [14] and an effective inhibition of agonist stimulated Pea5-HT_1_ was observed [29]. A more effective inhibition was measured for the mammalian 5-HT_7_ receptor antagonist, SB-269970, but no information regarding the possible effects of this antagonist is known from other arthropods. An effective dose-dependent inhibition was measured for prazosin, methiothepin and methysergide, although their IC_50_ values were in the micromolar range. All three are known to be non-selective antagonists for mammalian 5-HT receptors, including 5-HT_1_ receptors. At high concentrations (≥1 mM), they completely inhibited activation of Trica5-HT_1_ by 100 nM of 5-HT. When comparing dose-response curves of 5-HT in the presence of different concentrations of antagonist, all three antagonists behaved as competitive antagonists. Both the IC_50_ and the pA2 values indicated that prazosin was the most potent antagonist, followed by methiothepin and methysergide.

In conclusion, our data support previous findings that primary structures and signaling properties are well conserved between vertebrate and invertebrate receptors, yet pharmacological properties can differ significantly between both phyla, and even between different invertebrate species. The differences in pharmacological profiles of vertebrate and invertebrate receptors may be due to their large evolutionary distance. Selection during evolution most likely was based on receptor properties such as ligand binding and G protein coupling, but not on conservation of recognition sites for man-made, synthetic ligands. Furthermore receptor subtypes with specific pharmacological properties have evolved within the main classes of vertebrate receptors.

During the last decades, knowledge about 5-HT (and other biogenic amine) receptors has increased. However, comprehensive data on the pharmacology of insect or other invertebrate 5-HT receptors is still missing. Unequivocal identification and extensive characterization of all members constituting the invertebrate 5-HT receptor family are needed to establish a reliable classification scheme. Detailed pharmacological information for each 5-HT receptor subtype will also aid in functional *in vivo* studies and can be very useful for insect pest control.

## Supporting Information

Figure S1 **Nucleotide sequence of the** ***T. castaneum*** **5-HT_1_ receptor sequence (Trica5-HT_1_, accession no. KC196076**)**.** Inverted triangles indicate differences resulting in another amino acid between the current sequence derived from cloned cDNA and the annotated sequence from Beetlebase. Diamonds indicate silent mutations. The arrow indicates the splice site where a stretch of 75 residues is present in the Beetlebase sequence.
(TIF)

Table S1 Nucleotide sequences of primers for *T. castaneum* housekeeping genes.
(DOCX)
