# Supplementary figures and images for: Pharmacological Characterization of a 5-HT1-Type Serotonin Receptor in the Red Flour Beetle, Tribolium castaneum
Source: PLoS One. 2013 May 31;8(5):e65052. doi: 10.1371/journal.pone.0065052 (PMC3669024; doi:10.1371/journal.pone.0065052)

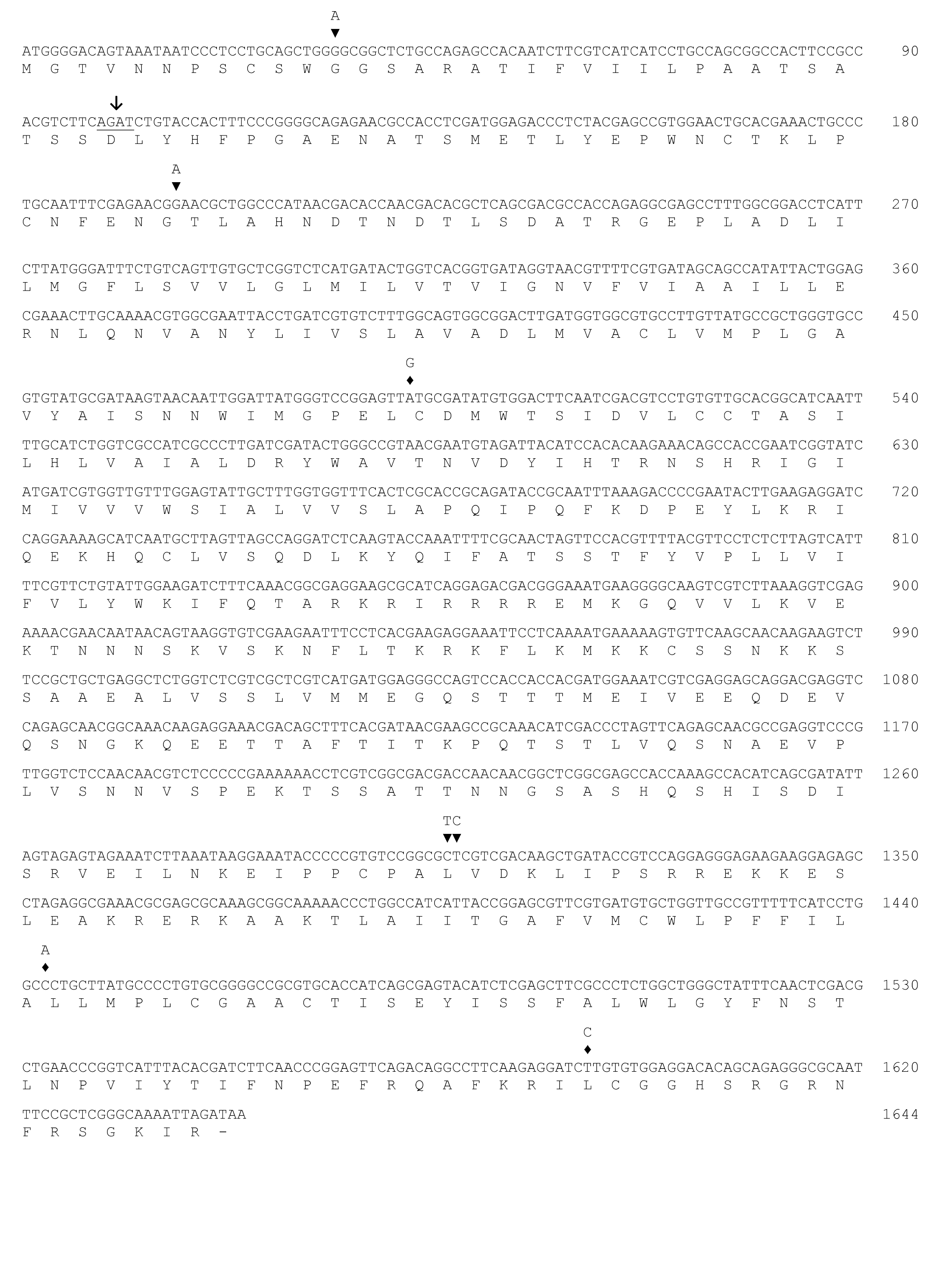

Supplement: Figure S1 — Nucleotide sequence of the T. castaneum 5-HT1 receptor sequence (Trica5-HT1, accession no. KC196076). Inverted triangles indicate differences resulting in another amino acid between the current sequence derived from cloned cDNA and the annotated sequence from Beetlebase. Diamonds indicate silent mutations. The arrow indicates the splice site where a stretch of 75 residues is present in the Beetlebase sequence. (TIF) [file pone.0065052.s001.tif]
