# Supplementary material for: Pharmacological Characterization of a 5-HT1-Type Serotonin Receptor in the Red Flour Beetle, Tribolium castaneum
Source: PLoS One. 2013 May 31;8(5):e65052. doi: 10.1371/journal.pone.0065052 (PMC3669024; doi:10.1371/journal.pone.0065052)
Supplement: Table S1 — Nucleotide sequences of primers for T. castaneum housekeeping genes. (DOCX) [file pone.0065052.s002.docx]

**Supplementary table 1:** Nucleotide sequences of primers for *T. castaneum* housekeeping genes.

| **Name** | **Forward primer** | **Reverse primer** |
| --- | --- | --- |
| **Tc-Act** | 5’-CGTGTCTTTTCAAACGTAAATACTAATCA-3’ | 5’-GCACATACCGGATCCATTGTC-3’ |
| **Tc- Ef1α** | 5’-TCGTAATCCGCCATGGGTAA-3’ | 5’-CGGAATCGTGACCAATC-3’ |
| **Tc-RpS3** [67] | 5’-ACCTCGATACACCATAGCAAGC-3’ | 5’-ACCGTCGTATTCGTGAATTGAC-3’ |
| **Tc-RPs13** | 5’-CCCCTTCACAAATAGGTGTCACA-3’ | 5’-TTGCCTGTTACGAACCGAACTT-3’ |
| **Tc-RPs18** [67] | 5’-CGAAGAGGTCGAGAAAATCG-3’ | 5’-CGTGGTCTTGGTGTGTTGAC-3’ |
| **Tc-RP49** | 5’-TGGCAAACTCAAACGCAACT-3’ | 5’-AGCGCCTACGAACCCTGTT-3’ |
| **Tc-Ubq** | 5’-AGATCCTCCCGCACAATGTT-3’ | 5’-GGCTTGCCAGTGAAATAAATCAT-3’ |
